# Epidemiological features of tuberculosis in the Middle East and North Africa from 1990 to 2019: results from the global burden of disease Study 2019

**DOI:** 10.4314/ahs.v23i3.43

**Published:** 2023-09

**Authors:** Mehdi Moradinazar, Zienab Mohseni Afshar, Uosef Ramazani, Mohammad Shakiba, Maria Shirvani, Sara Darvishi

**Affiliations:** 1 Behavioral Disease Research Center, Kermanshah University of Medical Sciences, Kermanshah, Iran

**Keywords:** Tuberculosis, disability, burden of disease, DALY, MENA

## Abstract

**Introduction:**

Tuberculosis (TB) is a preventable and curable disease, although, it still causes more than one million deaths annually. Therefore, the aim of this study was to measure the epidemiological status and the burden of TB in the Middle East and North Africa (MENA) countries.

**Methods:**

The study population included 21 countries in the MENA region, covering a population of about 400 million. The Global Burden of Disease (GBD) 2019 database was used. The case definition comprises all forms of TB, containing pulmonary and extra pulmonary TB, which are bacteriologically approved or clinically diagnosed. The prevalence, incidence, death, and the disability-adjusted life years (DALYs) rates per 100,000 people for all national locations by standardized age rates (ASR) were measured.

**Results:**

In 2019, Afghanistan had the highest TB-related incidence 85.09 (95% UI, 73.69_98.46), death 21.91 (95% UI, 13.44_29.78), and DALYs rate 695.21 (95% UI, 454.34_939.49). The highest prevalence rates of TB were in Egypt 28935.42 (95% UI, 26125.54_32251.01). The highest TB-related DALYs rate was attributed to alcohol use, high fasting plasma glucose, and smoking were related to Tunisia, Qatar, and Lebanon, respectively. Between 1990 and 2019, TB- related incidence, prevalence, death, and DALYs rate have decreased by 53%, 42.19%, 76.20%, and 75.95% in MENA region, respectively.

**Conclusion:**

TB has continued to decrease in prevalence, incidence, death, and DALYs rates in the MENA region, although, nowadays with the COVID-19 pandemic, societies may face more challenges for TB prevention, detection, treatment, and rehabilitation.

## Introduction

Tuberculosis (TB) is a preventable and curable disease, although, it causes more than one million deaths annually. 98% of TB deaths occur in less developed countries affecting most adults in the productive years of their life 1. In fact, deaths from TB are more than any other infectious disease throughout history. Approximately, a quarter of the world's population is infected with TB^2^. Evidences have shown that global deaths from TB are as twice as deaths from plague, malaria, cholera, smallpox, influenza, and acquired immunodeficiency syndrome (AIDS) during the last two centuries 3, 4.

Based on the global reports, the incidence rate of TB has decreased significantly in the recent years, although, there were about 10 million infected patients (range: 8.9 to 11 million) in 2019. The absolute numbers of TB deaths among HIV-negative people have been estimated to decrease by 27% during 2000 to 2019, from 1.7 million to 1.2 million.^5^ The TB deaths among HIV-positive people have also decreased from 678,000 deaths in 2000 to 208,000 in 2019 4, 6

Annually, numerous measures are taken to reduce and control the TB across the world 7. For example, the global treatment success rate of TB increased from 81% in 2016 to 85% in 2017 5, 8. TB may infect anyone anywhere, although, it is not equally distributed around the world 9. Therefore, identifying its various epidemiological aspects, including the prevalence, incidence, mortality, DALY, and trend is very crucial for policy-makers 8, 10.

Generally, health authorities should be aware of their country's health and compared it with other countries with similar socioeconomic conditions. In Middle East and North Africa (MENA) region, most health determinants are similar, however, there is a significant discrepancy in health and disease index among these countries 11. On the other hand, the MENA region have the highest incidence of TB due to parameters such as war and conflict, lack of proper health care infrastructure, financial crises, water crisis, natural disasters, migration and displacement. So that TB is known as a leading kill among young women in Africa.

Global Burden of Disease (GBD) studies are unique to estimate incidence, prevalence, death, and DALYs of diseases for all regions. GBD studies longitudinally report disease burden and create an opportunity to compare countries and explain the pattern of diseases. These studies set priorities, present other country's health experiences, help with resource allocation, and assist to tailor effective health interventions 12, 13. Thus, assessing the TB epidemiology in the MENA region may encourage policymakers to plan, establish, and develop TB prevention programs 14.

The aim of this study was to provide the epidemiological status and the burden of TB in the Middle East and North Africa (MENA) countries from 1990 to 2019. The results of the present study may help to design the targeted strategies for TB prevention tailored to various countries.

## Methods

### Geographical location and population

We selected two world regions including the Middle East and North Africa. MENA region, with 21 countries consisting of Afghanistan, Algeria, Bahrain, Egypt, Iran, Iraq, Jordan, Kuwait, Lebanon, Libya, Morocco, Palestine Oman, Qatar, Saudi Arabia, Sudan, Syria, Tunisia, Turkey, United Arab Emirates (UAE), and Yemen, covers a population of nearly 400 million 15.

### Data collection and quality control

The latest data refresh from the Global Burden of Disease (GBD) (the 2019 update) was used in the current study. The data of GBD estimates the incidence, prevalence, mortality, and DALYs of 369 diseases and injuries, and 84 risk factors in terms of location, age, and sex in 204 countries and regions (https://vizhub.healthdata.org/).

This dataset, as a scientific and systematic effort, contains annual reports from 1990 to 2019 for TB in all countries and regions. The methodological details of GBD 2019 have been reported elsewhere 16-18. The GBD determined the burden of TB in terms of incidence, prevalence and mortality using information gathered through surveillance systems (case notifications and death registrations), special studies (including surveys of the prevalence of TB), mortality surveys, inventory studies of under-reporting of detected TB, in-depth analysis of surveillance, household surveys, censuses, expert opinion and consultations with countries, and other data 19. In the GBD system, modelling for all countries is based on the quality and accessibility of data. Moreover, the same method was used for each country and time in all countries of the MENA Region, to achieve reliable and valid comparisons between various locations and years.

Due to public availability to the data of GBD, ethics approval and informed consent were not required.

### Definition of TB

TB, as an infectious disease, is caused by bacteria (Mycobacterium tuberculosis) that mostly affect the lungs. The case definition comprises all forms of TB, containing pulmonary and extra pulmonary TB, which are bacterio-logically approved or clinically diagnosed. At GBD 2019, TB includes all encoded diagnoses including A10–19.9, B90-90.9, K67.3, K93.0, M49.0, and P37.0 according to the 10th edition of the International Classification of Diseases (ICD-10) 20, 21.

GBD study evaluated the different risk factors attributable to TB disorders-related DALYs consisting of high fasting plasma glucose, smoking, and alcohol use 18.

Further information is available at http://vizhub.health-data.org/gbd-compare/.

### DALY calculation

The rates in GBD are standardized based on the total world population. DALYs index was calculated to compare different countries. DALYs are the sum of the years of life lost (YLLs) and the years lived with disability (YLDs) 22, 23. YLLs are counted as the product of the estimated number of deaths and a standard life expectancy at the age of death. YLDs are computed by multiplying the prevalence of individual consequences of the disease, by their corresponding disability weights, which quantify the severity of consequences as a number between 0 (full health) and 1 (death). Details of data sources and estimation methods have been published here 16, 24. Also, the age-standardized rate (ASR), age-standardized incidence rate, age-standardized prevalence rate, and age-standardized death rate in 100,000 people were reported 25, 26.

**Figure 1:**
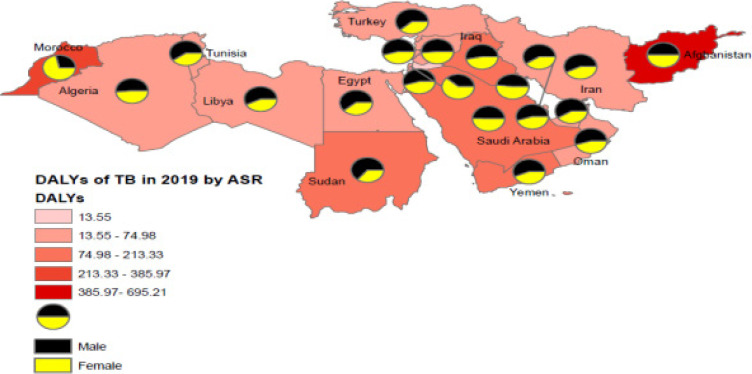
Comparison of the DALY of tuberculosis according to ASR between the sexes in 2019 in 100,000 population

Moreover, all data were extracted from GBD (https://vizhub.healthdata.org/). In general, the external validity of GBD was evaluated by performing cross-validation on a limited number of sequelae due to the computational time and complexity of this analysis. Then, we analysed the data based on the study's objectives e.g., age groups, risk factors, gender and etc. Also, all estimates were reported with 95% uncertainty intervals (UI). Moreover, all analyses and figures were applied by Microsoft Office Excel 2016. Also, ArcGIS 10.7.1 was used to prepare Figure 1.

## Results

In 2019, the highest age-standardized incidence rate of TB was in Afghanistan 85.09 (95% UI, 73.69_98.46) and Morocco 79.51 (95% U I, 67.41_93.97). The country of Jordan 4.83 (95% UI, 4.08_5.66) and Palestine 6.58 (95% UI, 5.68_7.64) has the lowest incidence rate of TB in 2019. The incidence rate of TB decreased by 53% in the MENA region from 1990 to 2019 (55.97 to 26.29), also the average global incidence rate of TB decreased by nearly 38% (172.55 to 106.71) between 1990 and 2019. In all countries of MENA region, the incidence rate of TB was lower than the global average.

In 2019, the highest age-standardized prevalence rates of TB were in Egypt 28935.42 (95% UI, 26125.54_32251.01), Lebanon 25899.77 (95% UI, 23003.04_29200.76), and Iran 25364.71 (95% UI, 22803.14_28334.37). The country of Jordan 2663.11 (95% UI, 2309.41_3097.7) and Algeria 9203.71 (95% UI, 8066.3_10584.59) had the lowest prevalence rate of TB in 2019. The prevalence rate of TB decreased by 42.19% in the MENA region from 1990 to 2019 (33401.06 to 19309.2), and also the average global prevalence rate of TB decreased by nearly 26.34% (31338 to 23085.13) between 1990 and 2019.

In 2019, the highest age-standardized death rates of TB were in Afghanistan 21.91 (95% UI, 13.44_29.78) and Morocco 13.63 (95% UI, 10.12_25.56). The country of Jordan 0.53 (95% UI, 0.44_0.64) had the lowest death rate of TB in 2019. The death rate of TB decreased by 76.20% in the MENA region from 1990 to 2019 (14.41 to 3.43), and also the average global death rate of TB decreased by nearly 63.48% (40.09 to 14.64) between 1990 and 2019. In 2019, the death rate of TB in the countries of the MENA region except for Afghanistan 21.91(95% UI, 13.44_29.78) was less than the average global death rate 14.64 (95% UI, 13.39_16.03).

In 2019, the highest age-standardized DALY rates of TB were in Afghanistan 695.21 (95% UI, 454.34_939.49) and Morocco 385.97 (95% UI, 290.69_694.4). The country of Jordan 13.55 (95% UI, 11.45_16.01) had the lowest DALY rate of TB in 2019 Table 1.

**Table 1:** Comparing the burden of tuberculosis in the world with the burden of disease in the Middle East and North African countries (MENA) from 1990 to 2019 by ASR

Country	Year	Incidence Per100.000 (95%CI)	Prevalence Per100.000 (95%CI)	Death Per100.000 (95%CI)	DALYs Per100.000 (95%CI)
Afghanistan	1990	170.85(150.27_193.2)	34139.91(31119.06_37180.4)	58.33(34.31_78.37)	2091.07(1264.4_2708.59)
	2019	85.09(73.69_98.46)	20972.33(18597.46_24034.49)	21.91(13.44_29.78)	695.21(454.34_939.49)
Algeria	1990	47.86(41.84_54.96)	21679.93(19380.98_24566.48)	7.83(6.01_9.62)	236.97(188.9_285.93)
	2019	17.23(14.92_20.05)	9203.71(8066.3_10584.59)	1.53(1.25_1.88)	42.94(35.6_51.79)
Bahrain	1990	41.78(36.31_47.98)	27352.41(24453.59_30610.01)	7.2(6.11_8.22)	181.46(157.48_207.45)
	2019	16.69(14.28_19.49)	11893.82(10468.88_13651.7)	1.65(1.33_2)	40.71(33.55_48.56)
Egypt	1990	23.62(21.01_26.5)	42572.36(39785.11_45229.29)	4.57(4.2_4.96)	190.63(176.04_207.56)
	2019	13.61(11.93_15.7)	28935.42(26125.54_32251.01)	1.23(0.9_1.9)	51.9(39.67_73.11)
Global	1990	172.55(150.96_197.79)	31338(28650.76_34205.22)	40.09(36.57_43.34)	1585.11(1454.22_1710.74)
	2019	106.71(93.96_121.94)	23085.13(20993.95_25380.06)	14.64(13.39_16.03)	590.42(536.85_646.42)
Iran	1990	20.83(18.35_23.72)	27669.63(25084.29_30350.24)	4.81(3.73_5.69)	156.88(126.99_178.13)
	2019	12.29(10.71_14.1)	25364.71(22803.14_28334.37)	1.17(1.07_1.32)	33.72(31.26_37.3)
Iraq	1990	78.76(69.11_89.39)	30168.75(27209.6_33249.9)	15.45(11.07_19.65)	552.5(417.6_684.26)
	2019	34.02(29.42_39.54)	15189.06(13322.49_17483.55)	3.87(3.07_4.84)	124.54(98.44_155.5)
Jordan	1990	14.76(12.6_17.26)	6458.01(5656.08_7429.6)	3.51(2.7_4.16)	89.17(71.57_102.77)
	2019	4.83(4.08_5.66)	2663.11(2309.41_3097.7)	0.53(0.44_0.64)	13.55(11.45_16.01)
Kuwait	1990	39.35(33.67_45.77)	20034.02(17673.91_22842.32)	5.51(4.69_6.5)	141.44(123.76_160.75)
	2019	17.37(14.97_20.28)	10048.37(8840.19_11582.95)	1.19(0.95_1.46)	29.89(24.82_35.89)
Lebanon	1990	34.13(29.41_39.57)	10318.51(9103.34_11926.66)	4.39(3.08_5.79)	142.32(106.57_181.52)
	2019	12.01(10.32_14.06)	25899.77(23003.04_29200.76)	0.8(0.55_1.48)	29.53(21.51_47.09)
Libya	1990	29.98(26.01_34.7)	27370.65(24324.9_30722.8)	4.49(3.32_5.74)	145.96(113.48_179.25)
	2019	15.11(13.02_17.61)	14796.49(12951.05_16944.51)	1.63(1.09_2.12)	51.47(36.61_65.63)
MENA	1990	55.97(49.68_62.58)	33401.06(30975.33_35744.26)	14.41(11.18_17.01)	486.32(397.38_556.75)
	2019	26.29(22.71_30.5)	19309.2(17281.95_21770.14)	3.43(2.86_4.23)	116.98(96.79_140.92)
Morocco	1990	162.21(141.83_183.26)	33714.32(30552.72_36869.13)	47.19(34.96_53.89)	1455.29(1207.51_1661.13)
	2019	79.51(67.41_93.97)	17488.74(15395.53_20079.38)	13.63(10.12_25.56)	385.97(290.69_694.4)
Oman	1990	43.38(37.47_49.87)	26451.26(23596.05_29681.64)	7.12(4.76_9.78)	205.05(146.57_270.35)
	2019	14.75(12.7_17.07)	11563.61(10172.56_13339.89)	1.23(0.95_1.58)	32.74(26.35_39.67)
Palestine	1990	17.7(15.25_20.51)	27493.7(24440.03_30755.42)	4.37(2.44_8.19)	134.65(87.46_231.82)
	2019	6.58(5.68_7.64)	14713.28(12951.55_16987.87)	1.14(0.85_1.4)	32.29(24.94_39.05)
Qatar	1990	37.77(32.8_43.36)	26211.56(23243.71_29355.98)	5.87(4.42_7.29)	159.54(126.22_194.26)
	2019	12.99(11.19_15.06)	10493.28(9257.18_12065.03)	1.4(1.08_1.79)	30.12(24.16_37.41)
Saudi Arabia	1990	97.71(87.36_108.25)	40463.29(38336.25_42691.94)	35.87(22.33_59.54)	835.84(536.32_1329.45)
	2019	42.53(36.55_49.81)	16126.71(14198.9_18479.3)	7.16(5.73_9.24)	186.94(150.78_236.91)
Sudan	1990	66.14(58.5_74.7)	35873.86(32776.39_38843.87)	22.96(13.37_33.29)	840.49(527.64_1159.63)
	2019	31.49(27.2_36.42)	20193.09(17869.97_23156.27)	5.21(3.03_7.65)	167.38(103.76_238.4)
Syria	1990	24.09(21.04_27.84)	29629.54(26557.64_32807)	3.03(2.31_3.65)	133.53(108.1_158.07)
	2019	9.41(8.06_11.04)	14058.01(12389.76_16243.54)	0.71(0.53_0.92)	28.11(22.12_35.27)
Tunisia	1990	32.74(28.44_37.84)	26223.38(23298.21_29439.18)	4.31(3.04_5.61)	140.34(109.93_170.51)
	2019	14.03(12.01_16.26)	12083.65(10641.55_13972.89)	1.04(0.72_1.46)	32.47(24.56_42.48)
Turkey	1990	51.33(45.33_57.51)	37316.15(34320.52_40257.08)	10.44(8.74_12.19)	409.64(350.93_480.52)
	2019	16.96(14.57_19.73)	16287.95(14354.44_18809.56)	0.97(0.78_1.2)	37.48(30.9_45.03)
UAE	1990	42.36(37.71_47.1)	39418.56(36748.45_41957.15)	8.88(5.92_12.42)	251.37(176.97_341.15)
	2019	21.88(18.93_25.36)	24646.16(21900.28_27851.42)	2.39(1.17_3.48)	74.98(43.22_105.36)
Yemen	1990	61.4(54.57_69.1)	35543.26(32391.87_38631.21)	21.39(13.44_35.9)	712.79(484.78_1108.66)
	2019	34.23(29.71_39.43)	21915.12(19462.28_24982.29)	6.92(4.81_10.04)	213.33(154.65_306.84)

In MENA, DALYs rate of TB in women 121.41 (95% UI, 96.54-159.50) was higher comparing men 112.96 (95% UI, 91.58-138.95). Also, Afghan women 697.49 (95% UI, 297.48-990.13) and Moroccan women 550.65 (95% UI, 401.18-1152.28) had the highest DALYs rate of TB in the region Fig. 1.

**Figure 2:**
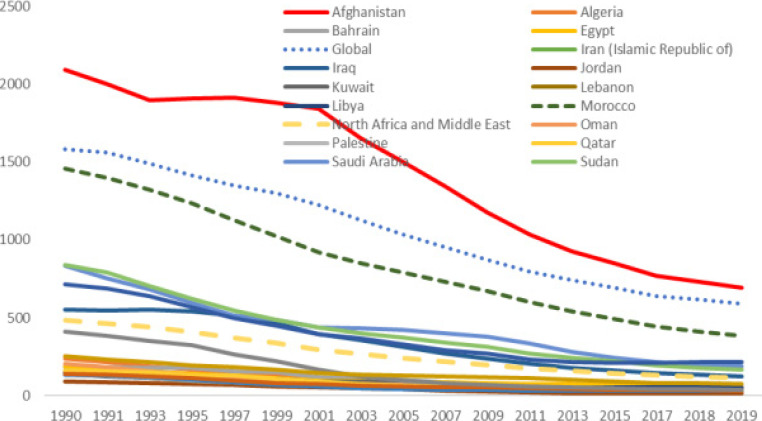
The DALY rate of tuberculosis by ASR in the MENA countries and in the world from 1990 to 2019

As have been shown in Figure 2, there was a decreasing trend in DALYs rate between 1990 and 2019 among the countries of the MENA region. Global DALYs rate have decreased by nearly 62.75% from 1990 1585.11 (95% UI, 1454.22_1710.74) to 2019 590.42 (95% UI, 536.85_646.42). In MENA, average DALYs rates have decreased by nearly 75.95% from 1990 486.32 (95% UI, 397.38_556.75) to 2019 116.98 (95% UI, 96.79_140.92). Between 1990 and 2019, the highest decrement of the TB -related DALYs with a 91% decrement was related to Turkey (409.64 to 37.48). Moreover, the lowest decrement of the TB -related DALYs with a 67% decrement was related to Afghanistan (2091.07 to 695.21) Fig. 2.

**Figure 3:**
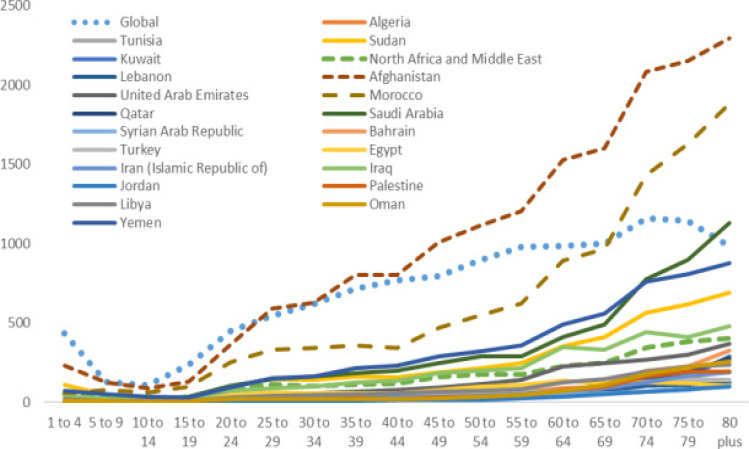
The age trend The DALY rate of tuberculosis in both sexes in the world and MENA countries in 2019

In 2019, the DALY rate of TB in the countries of the MENA region except for Afghanistan 695.21 (95% UI, 454.34_939.49) was less than the average global DALY rate 590.42 (95% UI, 536.85_646.42). However, the DALY of ages of 65 and more in Morocco was found to be higher than the global average in this age group. The lowest DALY rate was observed among people aged 5-19 years Fig. 3.

**Figure 4:**
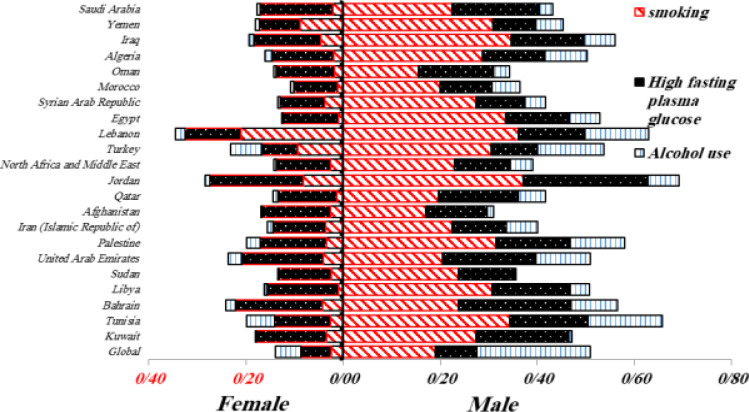
A percentage of total DALY related to Tuberculosis infection by ASR in both sexes attributed to the risk factors of Smoking, High fasting plasma glucose and Alcohol use in 2019

The highest TB-related DALYs rate was attributed to Alcohol use were related to Tunisia. The highest DALYs rate attributed to High fasting plasma glucose was related to the Qatar. The highest percentage of total DALY attributed to smoking was related to Lebanon. Overall, smoking had the greatest impact on the DALY in all MENA countries. However, alcohol use had greatest impact on the global DALY rate Fig.4.

## Discussion

In the MENA region, the incidence rate of TB has decreased by 53.0%, the average prevalence rate has decreased by 42.19%, the average death rate has decreased by nearly 76.20%, and the average DALY rate has decreased by 75.95 from 1990 to 2019. This decreasing trend in the MENA region was higher than the global average. The availability of treatment and knowledge about transmission and prevention can be strong reasons to decrease the prevalence and incidence of TB in 2019. Although the burden of the TB has decreased compared to the previous years, it still remains the most important infectious disease worldwide, therefore, it should not be ignored and the communities must increase their efforts to improve the TB diagnosis and treatment.

According to WHO reports, India, Indonesia, and China have been ranked first to third in the world in terms of the burden of TB^6^. The highest TB- related incidence, death, and DALYs in the MENA region belong to Afghanistan and Morocco. In 2019, the TB- related death and DALYs rate in the Afghanistan was higher than the average global death and DALYs rate. The highest prevalence rates of TB by ASR were in Egypt in 2019 which was higher than the average global prevalence rate. Alarmingly high incidence, death, and DALYs rates of TB were reported in Afghanistan may be driven by the prolonged war in this country. Prolonged war has prevented effective implementation of TB treatment and control strategies, which leads to high TB incidence rate and subsequent high death and DALYs rates. In Morocco, TB remains the most common infectious cause of death after neonatal disorders and low respiratory infections. Of course, this may be due to lifestyle, personal hygiene, nutritional status, and poverty 27.

In Afghanistan, TB- related DALYs rate have decreased by nearly 11.80% from 1990 to 2001 (2091.07 to 1844.42). Between 2001 and 2019, TB- related DALYs rate have decreased by 62.31% in Afghanistan. There are several causes for this declining trend. The most important reason is the National TB Programme (NTP), which was re-implemented in Afghanistan in 2001; it provided services such as improving the primary care, progressing the diagnosis and treatment strategies. Overall, it is clear that NTP could gradually reduce the burden of TB in Afghanistan. Afghanistan's NTP is an example of public health programs should be implemented effectively in vulnerable countries 27-29.

Jordan has the lowest prevalence, incidence, death, and DALYs rates of TB. This may be due to the Jordanian tuberculosis program reached the goal of the Millennium Development Goal for tuberculosis reduction in 2011 and was preparing to shift to tuberculosis elimination. Although, elimination planning has been delayed due to the Syrian crisis.

The result of the present study indicated that the TB-related DALY increased with aging in these 21 countries. However, the lowest DALY rate was observed among people aged 5-19 years. The evidences indicated that in high burden countries, the prevalence of TB peaked in early adulthood and affected people with economic productivity; however, in low burden countries, TB prevalence was peaked among the elderly people^5^,^30^.

We found that the DALYs rate of TB in women was higher compared to men. Conversely, previous studies found that men had higher TB-related DALY than women^2^,^31^, ^32^; however, WHO reported that the burden of TB was relatively similar among women and men recently^6^. In fact, the potential reasons why women had higher TB-related DALYs may be due to sex discrimination and bias, particularly in developing countries, women are often dependent to men financially, and in some societies the decision to seek treatment or access to diagnostic services for women is made by men 33. Therefore, further studies are recommended to determine the effect of gender on the burden of TB in MENA region.

The results of the present study showed that lifestyle factors including smoking, alcohol use, and elevated high fasting plasma glucose, especially in men, had the effect on the TB-related DALYs in the MENA region. In 2019, Tunisia had the highest DALYs in the region due to Alcohol use. The highest percentage of total DALYs attributed to the high fasting plasma glucose was related to Qatar. The highest percentage of total DALY attributed to smoking was related to Lebanon. Our findings showed that the highest impact of risk factors in TB-related DALY index in the MENA region was attributed to smoking.

A study by Lönnroth et al. reported that ethanol use (> 40gr per day) or alcohol use disorder increased the risk of TB by approximately three-fold (RR 2.94, 95% CI 1.89–4.59) comparing non-consumption or use below the specified threshold 34. A meta-analysis conducted by Imtiaz et al. indicated that in 2014, alcohol use has caused 169 721 TB deaths (95% CI 148 111–345 658) worldwide (2.35 deaths per 100 000 people, 95% CI 2.05–4.79). The African and South-East Asia regions exhibited the highest alcohol-attributable TB death, while the lowest impacts were featured in the Eastern Mediterranean Region 35. Overall, alcohol use and dosage were assessed as risk factors for TB by previous studies; they found a higher alcohol-attributable TB mortality at the highest levels of alcohol use^35^-^37^.

Prior evidences showed that smoking directly increased the risk of TB^38^,^39^. A study by Obore et al. sh owed that the smoking increased the risk of TB by nearly 2.5-fold. They reported that the Indoor air pollution (IAP) and the second-hand smoke also increased the risk of TB^32^. Furthermore, a study conducted in Morocco in 2018 found that smoking is the most important factor that affects the host defense against TB^40^. This may be due to that smoking reduce mucosal immunity in the respiratory tract and disrupt both innate and adaptive immune responses, consequently increases the risk of TB infection in smokers^41^,^42^.

## Strengths and limitations of the study

All limitations of the GBD study discussed elsewhere and fully apply to this study. By the way, we were not able to present burden estimates for pulmonary and extra pulmonary TB in detail, because these will be the subjects of separate studies. Another major limitation of this study is a lack of information on the other risk factors. Furthermore, the quality of the data collection system is different across countries, and the comparison of countries may be ambiguous. In July 2011, Sudan divided into two countries of North Sudan and South Sudan but was considered as a single country, showing another limitation of this study. Given GBD estimates are updated annually, the present limitations should be addressed.

The strengths of the present study were comparing the data of countries that approximately have the same information registration system and socio-demographic Index. Other strengths of the present study were the comprehensive estimations of TB burden as reported by prevalence, incidence, death, and DALYs between different countries from 1990 to 2019; hence, it can detect the strengths and weaknesses of the health care systems in different countries.

## Conclusion

TB has continued to decrease in prevalence, incidence, death, and DALYs rates in the MENA region, although, it still causes more than one million deaths annually. Given that most of the TB burden is advanced by modifiable risk factors, health care systems are crucially needed to set policies, allocate resources, tailor the educational intervention to modify unhealthy lifestyles such as smoking, alcohol use, and high fasting plasma glucose. Nowadays with the COVID-19 pandemic, societies may face more challenges for TB prevention, detection, treatment, and rehabilitation. In general, health policymakers can use the information reported in this study to develop their health planning.
